# Daytime Sleepiness Is Associated with Lower Cognitive Scores: The Look AHEAD Study

**DOI:** 10.14283/jarlife.2023.9

**Published:** 2023-06-26

**Authors:** K.M. Hayden, A. Anderson, A.P. Spira, M.-P. St-Onge, J. Ding, M. Culkin, D. Molina-Henry, A.H. Sanderlin, D. Reboussin, J. Bahnson, M.A. Espeland

**Affiliations:** 1 Department of Social Sciences and Health Policy, Wake Forest University School of Medicine, Winston-Salem, NC, USA; 2 Department of Biostatistics and Data Science, Wake Forest University School of Medicine, Winston-Salem, NC, USA; 3 Department of Mental Health, Johns Hopkins Bloomberg School of Public Health, Baltimore, MD, USA; 4 Department of Psychiatry and Behavioral Sciences, Johns Hopkins School of Medicine, Baltimore, MD, USA; 5 Johns Hopkins Center on Aging and Health, Baltimore, MD, USA; 6 Department of Medicine, Columbia University Irving Medical Center, New York, NY, USA; 7 Department of Internal Medicine, Gerontology and Geriatric Medicine, Wake Forest University School of Medicine, Winston-Salem, NC, USA; 8 Winston-Salem State University, Winston-Salem, NC, USA; 9 University of Southern California, Alzheimer’s Therapeutic Research Institute, San Diego, CA, USA; 10 Department of Biology, North Carolina Agricultural and Technical State University, Greensboro, NC, USA

**Keywords:** Sleep disorders, diabetes mellitus, type 2, cognition disorders, aging, obesity, overweight

## Abstract

**Background:**

Daytime sleepiness is common in older adults and may result from poor nighttime sleep due to sleep disordered breathing, fragmented sleep, or other sleep disorders. Daytime sleepiness may be associated with cognition in older adults.

**Objectives:**

We investigated the association between self-reported daytime sleepiness and cognitive function in the Look AHEAD clinical trial.

**Design:**

Observational follow-up of a randomized clinical trial of an intensive lifestyle intervention.

**Setting:**

Clinic.

**Participants:**

Participants (n=1,778) aged 45-76 years at baseline with type 2 diabetes and overweight or obesity.

**Interventions:**

Participants were randomized to an intensive lifestyle intervention for weight loss or a control condition of diabetes support and education.

**Measurements:**

Participants provided self-reported levels of daytime sleepiness at baseline and years 12-13. Cognitive function was assessed with a neurocognitive battery at years 12-13 and 18-20.

**Results:**

Participants who reported having frequent daytime sleepiness (often or always) performed significantly worse than others on the cognitive composite (-0.35; p-value=0.014) after controlling for covariates. When stratified by intervention arm, participants assigned to the intensive lifestyle intervention who reported often/always having daytime sleepiness performed worse on Digit Symbol Coding (-0.63; p-value=0.05) and Trail Making Part-B (-0.56; p-value=0.02) after controlling for covariates. Statistical interactions revealed associations between daytime sleepiness and the following covariates: race and ethnicity, APOE ε4 carrier status, baseline history of cardiovascular disease, and depression.

**Conclusions:**

Daytime sleepiness over ~13 years predicted poorer cognitive performance in older individuals who, by virtue of having diabetes and overweight/obesity, are at high risk for sleep disorders and cognitive impairment.

## Introduction

**D**aytime sleepiness is common in older adults (1, 2) and may signal health problems (3). Daytime sleepiness may result from sleep-disordered breathing, fragmented sleep or other sleep disorders (2), or from insufficient sleep, and it has been associated with comorbidities, medication use, and metabolic syndrome (4). The Cardiovascular Health Study showed associations between daytime sleepiness and incident cardiovascular disease (CVD) and mortality (3), and other studies have shown associations with cognitive decline (5, 6) and increased dementia risk (7, 8). Daytime sleepiness may signify disruption of circadian rhythms and may occur due to disorder in neural circuitry that may be affected in neurodegenerative disease including impairment in arousal systems (9). The locus coeruleus noradrenergic arousal system is associated with wakefulness and attention (10) and is affected early in the progression of neurodegeneration in Alzheimer’s disease (AD) (11). Self-reported excessive daytime sleepiness has been associated with later beta amyloid deposition, a pathological feature of AD, in the Baltimore Longitudinal Study of Aging (12).

Conditions associated with excessive daytime sleepiness and AD risk include obesity and diabetes (13). Among those with obesity and metabolic syndrome, heightened sympathetic activity can potentially cause fragmented sleep, leading to daytime sleepiness (14). Another factor, sleep-disordered breathing, could play a role, however daytime sleepiness among those with obesity or diabetes is not always associated with sleep-disordered breathing. Indeed, in a large study (n=16,583) of men and women, excessive daytime sleepiness was more strongly associated with greater body mass index (BMI), diabetes, and depression, than with sleep disordered breathing (13).

Previously in the Look AHEAD study, weight loss was shown to improve indices of sleep-disordered breathing and even remission of obstructive sleep apnea (OSA) (15), however daytime sleepiness was not studied. Look AHEAD provides the opportunity to evaluate the potential effects of a lifestyle intervention on daytime sleepiness and subsequent effects on cognitive function in a large, well-characterized cohort. Further, the large sample with cognitive assessments in Look AHEAD facilitates sub-analyses of gender differences and potential differences by APOE ε4 status, two important dementia risk factors, as well as subgroups for which we have found significant interactions in prior work (16-18) including age, baseline BMI, and baseline history of CVD. The objective of this analysis was to determine the degree to which self-reported daytime sleepiness was associated with the intervention and with cognition.

## Methods

The study design, methods (19), and CONSORT diagram (20) for Look AHEAD have been published. Briefly, Look AHEAD was a randomized controlled clinical trial of (n=5,145) participants aged 45-74 with diabetes and overweight/obesity. The trial was designed to determine whether intentional weight loss is appropriate for older adults with diabetes and overweight/obesity, with primary end points of fatal and nonfatal cardiovascular events. Eligibility criteria required that participants have BMI >25 kg/m^2^ (>27 kg/m^2^ if on insulin), glycated hemoglobin (HbA1c) <11%, systolic/diastolic blood pressure <160/100 mmHg, and triglycerides <600 mg/dl. Participants were required to demonstrate over a two-week run-in period, the ability to record daily, their diet and physical activity. Each participant met with a behavioral psychologist or interventionist to confirm that intervention requirements were understood and that participants did not have any competing life stressors that would impair adherence to the protocol. Study data were collected by certified, trained staff who were masked to intervention assignments (19). Participants were randomly assigned with equal probability to either the intensive lifestyle intervention (ILI) or diabetes support and education (DSE) arm of the trial. Enrollment and initiation of intervention delivery occurred between 2001 and 2004. Interventions continued until 2011, at which time participants were invited to join a follow-up observational study to determine the longer-term effects of the intervention on outcomes. The average intervention duration for participants in this study was 9.8 years [8.4-11.1y]. Local Institutional Review Boards approved the protocols and all participants provided written informed consent.

The ILI was a multidomain intervention including dietary modification and increased physical activity with a goal of inducing an average of ≥7% weight loss at one year and maintenance of weight loss over the course of the study (21). Participants in the ILI arm were given a daily calorie goal of 1200-1800 kcal based on initial weight. The diet specified <30% total calories from fat (<10% saturated fat) and a minimum of 15% total calories from protein. The physical activity goal was similar in intensity to brisk walking for at least 175 minutes/week. Participants randomized to the DSE condition were invited, but not required, to attend three group sessions/ year. Sessions focused on diet, physical activity, and social support (22). There were no specific instructions or goals for weight loss, physical activity, or dietary modification.

### Daytime Sleepiness

At baseline and during extended post-intervention follow-up (12-13 years later), participants were asked about daytime sleepiness: “How often do you feel excessively(overly) sleepy during the day.” Responses included never(1 day/month or less), sometimes(2-4 days/month), often(5-15 days/month), and almost always(16-30 days/month). Baseline and extended follow-up reports of daytime sleepiness were each and classified into three groups: 1) never, 2) sometimes, and 3) often or almost always.

### Cognitive Function

Cognitive assessments were conducted 1-4 times during follow-up during years 8-18 as part of the study follow-up protocol and participation of subsets of the cohort in ancillary studies (16). We used the most recent cognitive scores for the current evaluation (2018-2020). Staff were centrally trained and certified in administration of the standardized cognitive assessments and were masked to participant’s randomization status (23). The cognitive battery included the Rey Auditory Verbal Learning Test (RAVLT)(24), Digit Symbol Coding (DSC) (25), the Modified Stroop Color and Word Test (Stroop) (26), and the Trail Making Test Parts A&B (27). The Modified Mini-Mental Status Exam (3MS)(28) was used to assess global cognitive function. Test scores were standardized as z-scores which were averaged to derive a cognitive composite score (23). Trail Making Test scores were re-ordered so that higher values indicate better performance.

### Other measures

Staff collected demographic and clinical characteristics including age, gender, race and ethnicity, education level, and smoking status at baseline. Weight was measured with digital scales. Diabetes treatments (insulin, sulfonylureas, other) were recorded at baseline. Hypertension was defined by treatment or measured blood pressure >140/90 mmHg. Baseline CVD included self-report of myocardial infarction, heart bypass surgery, coronary artery bypass graft, carotid endarterectomy, lower leg angioplasty, aortic aneurysm, congestive heart failure, or stroke. The Beck Depression Inventory (BDI) (29) was assessed annually until year 14. Subsequently, the Patient Health Questionnaire-9 (PHQ-9) (30) was administered to assess depressive symptoms. We dichotomized depressive symptoms, with BDI scores of ≥11 and PHQ-9 scores of ≥5. APOE ε4 status, a dementia risk factor, was determined for participants who provided consent (80% of women versus 86% of men, p<0.001), using TaqMan genotyping (rs7412 and rs429358)(31).

### Analytic Design

Descriptive statistics were prepared by intervention group. Continuous variables were compared with t-tests and categorical variables compared with χ2 tests. Comparisons of continuous variables across three levels of daytime sleepiness were made using ANOVA. Categorical variables were derived to represent daytime sleepiness at baseline and follow-up 12-13 years later (2013-2014). The daytime sleepiness variable ranged from 1- 3 based on the categories as described above (i.e., never; sometimes; often/almost always). We compared individuals who were included in the analysis to those who were not included due to attrition or missing data.

Regression analysis assessed the association between daytime sleepiness in 2013-2014 and cognitive performance in 2018-2020 adjusting for: intervention arm, age, gender, race and ethnicity, education, baseline levels of daytime sleepiness, BMI, and hypertension. We adjusted for prior cognitive scores and depressive symptoms at baseline and as assessed concurrently with each cognitive measure. We did not use time-varying covariates for risk factors to allow the evaluation of the impact of the intervention. As prior work suggested heterogeneous effects of the intervention by subgroups (18, 32), we tested interactions by intervention arm, age (+/-65 at baseline), gender, race and ethnicity, APOE ε4 status, baseline BMI, and baseline history of CVD; we tested for an interaction by baseline depression as depression is associated with both cognition and daytime sleepiness.

## Results

**Figure 1.**
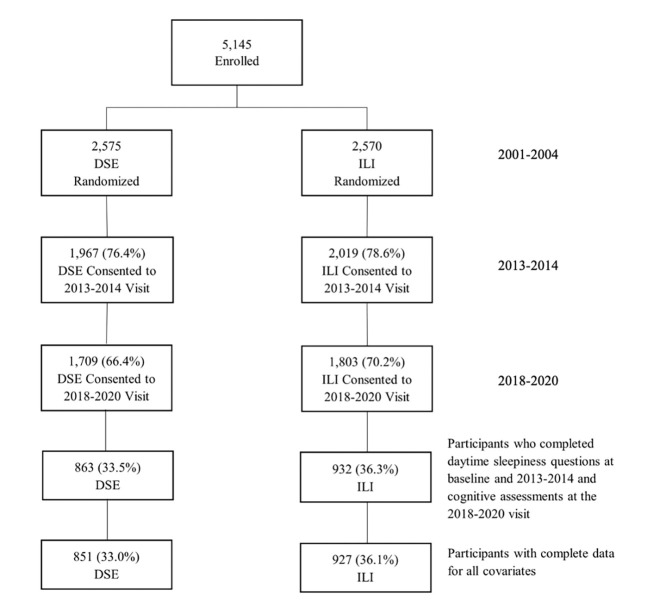
Sample Selection Flowchart

Participants who completed daytime sleepiness questions at baseline and proximal to the end of the intervention, and who completed cognitive evaluation at the most recent visit (n=1,778) were included in the analyses (Figure 1). A total of 3,367 participants were not included due either to being lost to follow-up or missing data for covariates of interest. These participants tended to be older, had lower levels of education, and included more APOE ε4 carriers; more of them had a baseline history of CVD, hypertension, insulin use, longer duration of diabetes, higher average HbA1c levels, and reported more depressive symptoms (Supplemental Table 1).

DSE=Diabetes Support and Education; ILI=Intensive Lifestyle Intervention

Among those included in the analysis, the average baseline age was 57 (standard deviation [SD] 6.3). The sample included more women (61%) than men, and was mostly White (63.9%) and highly educated (45.3% having college degree or higher). There were no significant differences by intervention arms among covariates listed in Table 1 except baseline BMI: the DSE group had a slightly higher average BMI (36.4 vs. 35.8 kg/m^2^) at baseline (p=0.02).

**Table 1.** Baseline characteristics of 1,778 Look AHEAD participants by Intervention Arm

Baseline Characteristics	Total	Treatment Arm		p-value
		DSE	ILI	
N	1778	851	927	
Age (SD)	57.2 (6.3)	57.1 (6.4)	57.3 (6.2)	0.55
45-54	680 (38.2%)	332 (39.0%)	348 (37.5%)	
55-64	931 (52.4%)	438 (51.5%)	493 (53.2%)	0.77
65-74	167 (9.4%)	81 (9.5%)	86 (9.3%)	
Gender (% female)	1085 (61.0%)	514 (60.4%)	571 (61.6%)	0.61
Race and Ethnicity (%)				
African American	263 (14.8%)	126 (14.8%)	137 (14.8%)	0.06
Non-Hispanic White	1136 (63.9%)	562 (66.0%)	574 (61.9%)	
Hispanic	255 (14.3%)	102 (12.0%)	153 (16.5%)	
Other	124 (7.0%)	61 (7.2%)	63 (6.8%)	
Education (%)				
<13 years	327 (18.4%)	146 (17.2%)	181 (19.5%)	0.27
13-16 years	646 (36.3%)	323 (38.0%)	323 (34.8%)	
>16 years	805 (45.3%)	382 (44.9%)	423 (45.6%)	
APOE ε4 carrier (%)	385 (21.6%)	173 (20.3%)	212 (22.9%)	0.19
Body Mass Index (SD)	36.1 (6.0)	36.4 (6.0)	35.8 (5.9)	0.02
25-29	262 (14.7%)	111 (13.0%)	151 (16.3%)	0.11
30-39	1103 (62.0%)	531 (62.4%)	572 (61.7%)	
≥40	413 (23.2%)	209 (24.6%)	204 (22.0%)	
History of CVD (%)	168 (9.5%)	80 (9.4%)	88 (9.5%)	0.95
Hypertension (%)	1434 (80.7%)	684 (80.4%)	750 (80.9%)	0.78
Insulin use (%)	255 (14.3%)	123 (14.5%)	132 (14.2%)	0.90
Diabetes duration, years (SD)	6.5 (6.4)	6.5 (5.8)	6.6 (6.9)	0.56
HbA1c (SD)	7.23 (1.15)	7.22 (1.13)	7.23 (1.16)	0.91
Smoking status (%)				
Current	80 (4.5%)	33 (3.9%)	47 (5.1%)	0.45
Former	787 (44.3%)	383 (45.0%)	404 (43.6%)	
Never	911 (51.2%)	435 (51.1%)	476 (51.4%)	
Depressive symptoms (%)				
BDI score <11	1570 (88.3%)	751 (88.3%)	819 (88.4%)	0.95
BDI score ≥11	208 (11.7%)	100 (11.7%)	108 (11.6%)	
Daytime sleepiness (%)				
Never	786 (44.2%)	362 (42.5%)	424 (45.7%)	0.29
Sometimes	581 (32.7%)	297 (34.9%)	284 (30.6%)	
Often	239 (13.4%)	111 (13.0%)	128 (13.8%)	
Almost Always	172 (9.7%)	81 (9.5%)	91 (9.8%)	

Abbreviations: APOE ε4=Apolipoprotein E gene, ε4 carrier status; BDI=Beck Depression Inventory; CVD=cardiovascular disease; DSE=diabetes support and education; ILI=intensive lifestyle intervention; SD=standard deviation.

Participant characteristics are reported in Table 2 by daytime sleepiness status at the 2013-2014 follow-up. A higher proportion of White participants reported often/ always having daytime sleepiness and more Hispanic participants reporting sometimes having daytime sleepiness (p<0.001). Those with higher baseline BMIs reported more frequent daytime sleepiness (p=0.02). A higher proportion of participants with hypertension reported having daytime sleepiness sometimes as opposed to never or often/always (p=0.006). Depressive symptoms were more common among those who reported often/always having daytime sleepiness (p<0.001).

**Table 2.** Baseline Characteristics of 1,778 Look AHEAD participants by Daytime Sleepiness at 2013-2014

Baseline Characteristics	Daytime Sleepiness			p-value
	Never	Sometimes	Often / Always	
N	762	627	389	
Age (SD)	57.4 (6.4)	56.9 (6.2)	57.5 (6.3)	0.21
45-54	289 (37.9%)	254 (40.5%)	137 (35.2%)	0.40
55-64	395 (51.8%)	319 (50.9%)	217 (55.8%)	
65-74	78 (10.2%)	54 (8.6%)	35 (9.0%)	
Gender (% female)	461 (60.5%)	395 (63.0%)	229 (58.9%)	0.39
Race and Ethnicity (%)				
African American	120 (15.8%)	99 (15.8%)	44 (11.3%)	<0.001
Non-Hispanic White	504 (66.1%)	363 (57.9%)	269 (69.2%)	
Hispanic	100 (13.1%)	101 (16.1%)	54 (13.9%)	
Other	38 (5.0%)	64 (10.2%)	22 (5.7%)	
Education (%)				
<13 years	137 (18.0%)	122 (19.5%)	68 (17.5%)	0.74
13-16 years	275 (36.1%)	234 (37.3%)	137 (35.2%)	
>16 years	350 (45.9%)	271 (43.2%)	184 (47.3%)	
APOE ε4 carrier (%)	164 (21.5%)	146 (23.3%)	75 (19.3%)	0.32
Body Mass Index (SD)	35.6 (5.9)	36.3 (6.0)	36.5 (5.9)	0.02
25-29	118 (15.5%)	94 (15.0%)	50 (12.9%)	0.31
30-39	484 (63.5%)	377 (60.1%)	242 (62.2%)	
≥40	160 (21.0%)	156 (24.9%)	97 (24.9%)	
History of CVD (%)	73 (9.6%)	49 (7.8%)	46 (11.8%)	0.10
Hypertension (%)	604 (79.3%)	530 (84.5%)	300 (77.1%)	0.01
Insulin use (%)	100 (13.1%)	88 (14.0%)	67 (17.2%)	0.17
Diabetes duration, years (SD)	6.2 (6.1)	6.6 (6.7)	7.0 (6.3)	0.13
HbA1c (SD)	7.26 (1.19)	7.20 (1.10)	7.20 (1.13)	0.58
Smoking status (%)				
Current	25 (3.3%)	39 (6.2%)	16 (4.1%)	0.09
Former	347 (45.5%)	263 (42.0%)	177 (45.5%)	
Never	390 (51.2%)	325 (51.8%)	196 (50.4%)	
Depressive symptoms (%)				
BDI score <11	703 (92.3%)	546 (87.1%)	321 (82.5%)	<.001
BDI score ≥11	59 (7.7%)	81 (12.9%)	68 (17.5%)	
Intervention Arm				
DSE	359 (47.1%)	306 (48.8%)	186 (47.8%)	0.82
ILI	403 (52.9%)	321 (51.2%)	203 (52.2%)	
Daytime sleepiness (%)				
Never	455 (59.7%)	230 (36.7%)	101 (26.0%)	<.001
Sometimes	207 (29.2%)	248 (39.6%)	126 (32.4%)	
Often	62 (8.1%)	91 (14.5%)	86 (22.1%)	
Almost Always	38 (5.0%)	58 (9.3%)	76 (19.5%)	

Abbreviations: APOE ε4=Apolipoprotein E gene, ε4 carrier status; BDI=Beck Depression Inventory; CVD=cardiovascular disease; DSE=diabetes support and education; ILI=intensive lifestyle intervention; SD=standard deviation.

**Figure 2:**
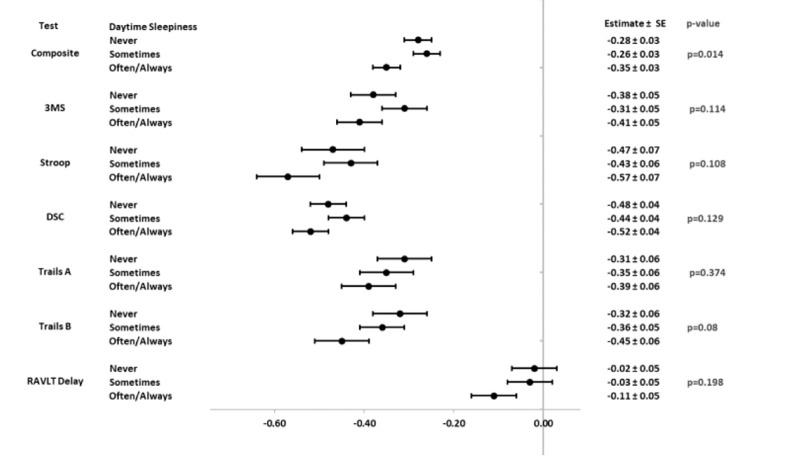
Cognitive performance (2018-2020) by daytime sleepiness (2013-2014)

Abbreviations: 3MS=Modified Mini-mental State Exam; DSC=Digit Symbol Coding; RAVLT Delayed=Rey Auditory Verbal Learning Test Delayed; Trails A=Trail Making Test Part A; Trails B=Trail Making Test Part B. *Models are adjusted for treatment arm, the 2013-2014 value of the outcome, depressive symptoms at baseline, 2013-2014, and 2018-2020 visits, and baseline values of daytime sleepiness, age, gender, race and ethnicity, education, BMI, and hypertension.

Figure 2 shows forest plots for least square means (±standard error) and p-values for associations between cognitive scores and frequency of self-reported daytime sleepiness. Models were adjusted for potential confounders including age, gender, race, ethnicity, education, randomization arm, and baseline values of BMI, hypertension, daytime sleepiness, and depressive symptoms. Models were also adjusted for 2013-2014 cognitive scores and concurrent assessments of depressive symptoms. Diabetes duration and smoking were considered but did not significantly contribute to the models and were dropped. Although the only statistically significant result is in the cognitive composite (p=0.014), forest plots demonstrate general dose-response effects such that participants reporting daytime sleepiness occurring often or always, had lower mean scores than those who reported lower levels of daytime sleepiness.

In sensitivity analyses, we re-fitted models without controlling for cognitive scores at years 13-14 and similar but stronger associations between daytime sleepiness groups and some cognitive scores emerged (Supplemental Table 2). The composite z-score was in the same direction but lost significance (p=0.06), while the DSC (p=0.01) and Trails B (p=0.05) became significant when we did not control for prior cognitive scores.

We tested interactions by intervention arm, age, gender, race and ethnicity, APOE ε4 status, baseline BMI, history of CVD, and depression as suggested by prior work in Look AHEAD(16-18) and the literature on daytime sleepiness(33), using a p-value threshold of p=0.10. Associations between daytime sleepiness and cognitive scores varied across intervention groups (Table 3) with significant findings among those in the ILI group on DSC (p=0.05) and Trails B (p=0.02) such that those reporting daytime sleepiness often/always performed worse than the other two sleep groups (i.e., never or sometimes). We show similar results, albeit with stronger effects in Supplemental Table 3 which does not include adjustment for prior cognitive scores.

**Table 3.** Cognitive Performance in 2018-2020 by Daytime Sleepiness at 2013-2014 Stratified by Intervention Arm [LS Mean (SE)]

Intervention Arm	Outcome	Daytime Sleepiness at 2013-2014			
		Never	Sometimes	Often / Always	p-value*
DSE	Composite z-score	-0.25 (0.04)	-0.22 (0.04)	-0.30 (0.04)	0.15
	3MS z-score	-0.32 (0.07)	-0.31 (0.07)	-0.42 (0.07)	0.26
	Stroop z-score	-0.43 (0.09)	-0.38 (0.09)	-0.57 (0.09)	0.10
	DSC z-score	-0.43 (0.06)	-0.36 (0.06)	-0.40 (0.06)	0.32
	Trails-A z-score	-0.25 (0.08)	-0.24 (0.08)	-0.30 (0.08)	0.76
	Trails-B z-score	-0.31 (0.07)	-0.27 (0.07)	-0.33 (0.08)	0.63
	RAVLT Delayed z-score	-0.05 (0.07)	-0.02 (0.07)	-0.08 (0.07)	0.65
ILI	Composite z-score	-0.31 (0.04)	-0.30 (0.04)	-0.40 (0.04)	0.08
	3MS z-score	-0.44 (0.08)	-0.32 (0.08)	-0.41 (0.08)	0.14
	Stroop z-score	-0.51 (0.09)	-0.49 (0.09)	-0.58 (0.09)	0.68
	DSC z-score	-0.51 (0.05)	-0.51 (0.05)	-0.63 (0.05)	0.05
	Trails-A z-score	-0.36 (0.09)	-0.45 (0.08)	-0.48 (0.09)	0.31
	Trails-B z-score	-0.34 (0.08)	-0.47 (0.08)	-0.56 (0.08)	0.02
	RAVLT Delayed z-score	-0.01 (0.07)	-0.06 (0.07)	-0.15 (0.07)	0.18

Abbreviations: 3MS=Modified Mini-mental State Exam; DSC=Digit Symbol Coding; RAVLT Delayed=Rey Auditory Verbal Learning Test Delayed; Trails A=Trail Making Test Part A; Trails B=Trail Making Test Part B. *Stratified models are adjusted for the 2013-2014 value of the outcome, depressive symptoms at baseline, 2013-2014 and 2018-2020 visits, and baseline values of daytime sleepiness, age, gender, race and ethnicity, education, BMI, and hypertension.

There were no significant interactions by age, gender, or baseline BMI. However, there were significant interactions between race and ethnicity categories and daytime sleepiness on nearly all our measures (shown in Supplemental Table 4). The trend across scores suggests lower performance on the composite (p=0.01), Stroop (p=0.03), DSC (p=0.02), Trails A (p=0.09) and Trails B (p=0.08), and RAVLT Delayed (p=0.07) with greater levels of daytime sleepiness. In most cases, African American and White participants demonstrated worse scores with more self-reported daytime sleepiness (sometimes or often/always). However, some apparent inconsistencies could be due to small numbers for groups including Hispanic participants and ‘other’ (which includes American Indian/Native American, mixed race, and others).

Interactions were found between daytime sleepiness and APOE ε4 status on cognitive scores on Trails A (p=0.05) and RAVLT Delayed (p=0.08) such that those who reported more daytime sleepiness and have one or more APOE ε4 allele(s), tended to perform worse than those without APOE ε4 allele(s). These interactions should be interpreted with caution however, because the number of participants with one or more APOE ε4 allele(s) is relatively small.

An interaction between baseline history of CVD and daytime sleepiness was apparent on the Stroop, with worse performance corresponding to higher levels of daytime sleepiness among those without a baseline history of CVD. Those who reported a baseline history of CVD and no daytime sleepiness performed worse than all the other groups (never: LS Mean= -0.69; p<0.01) on the Stroop. On the Trail Making Test Part A, participants with a baseline history of CVD demonstrated a trend toward worse performance with greater levels of self-reported daytime sleepiness (often/always: LS Mean=-0.68; p<0.08). On the Trail Making Test Part B, a similar trend was apparent with participants who had a baseline history of CVD performing worse with greater levels of daytime sleepiness (often/always: LS Mean=-0.63; p<0.09).

Finally, because there are established associations between depression and cognitive performance (34) as well as between depression and daytime sleepiness (35, 36), we tested for interactions between daytime sleepiness and depressive symptoms. Those with BDI score≥11 and no self-reported daytime sleepiness performed worse on DSC (never: LS Mean=-0.59) than those scoring <11; while those with BDI<11 and daytime sleepiness often or always performed nearly the same (LS Mean=-0.58). On Trails A, participants with BDI≥11 and reported daytime sleepiness sometimes (LS Mean=-0.59) and participants with BDI<11 reporting daytime sleepiness often/always (LS Mean=-0.43) performed worse than others (p<0.01). On Trails B the same pattern emerged where those with BDI≥11 and reported daytime sleepiness sometimes (LS Mean=-0.44) and participants with BDI<11 reporting daytime sleepiness often/always (LS Mean=-0.53) performed worse than others (p=0.02).

## Discussion

We sought to test the degree to which self-reported daytime sleepiness was associated with the Look AHEAD intervention and cognitive scores. Participants who self-reported daytime sleepiness often or always in 2013-2014 performed significantly worse than those who reported sometimes or never having daytime sleepiness on the cognitive composite. Individual tests suggested a dose-response relationship, with greater levels of daytime sleepiness associated with worse performance. We further stratified by intervention arm, showing poorer scores on executive function tests were driven by the ILI group. This is aligned with prior reports showing no long-term cognitive benefit from the intervention (16). Randomization to ILI was not associated with self-reported daytime sleepiness, although it is feasible that any benefits accrued as a result of the intervention were subsequently lost over time.

To further probe drivers of these associations, we tested interactions based on prior work (16-18) and found differences by racial and ethnic groups on most tests, with African American and White participants demonstrating more consistent associations between greater levels of daytime sleepiness and poor performance on various tests compared to participants from Hispanic and Other (American Indian/Native American, mixed race, and others) groups. Participants with one or more APOE ε4 allele(s) and greater levels of daytime sleepiness performed worse on Trails A and RAVLT than non- APOE ε4 carriers. Poorer cognitive performance was observed among those with more frequent daytime sleepiness and a history of CVD compared to those with less frequent daytime sleepiness and no history of CVD. These results support earlier findings in Look AHEAD that suggested that participants reporting a baseline history of CVD experienced fewer cognitive benefits compared to others in the cohort (16). This result is also not surprising as CVD is associated with daytime sleepiness (37). Finally, tests for interactions with depressive symptoms showed consistent associations between higher daytime sleepiness levels and lower cognitive function among those reporting low levels of depression. However, among participants with greater levels of depressive symptoms, associations between daytime sleepiness and cognition were complex. This may be due to the bidirectional association between depression and disrupted sleep patterns (38).

Daytime sleepiness has been associated with adverse health outcomes in addition to CVD (37), including cognitive decline (5), cognitive impairment and dementia (7), as well as amyloid deposition (12, 39). In our study, daytime sleepiness was associated with poorer scores on the cognitive composite overall; on DSC and Trails B (executive function) among participants in the ILI group; and poorer scores on the Stroop, Trails A, and Trails B among those with a baseline history of CVD.

Our study has some limitations and strengths to note. Cognitive function was not measured at baseline as it was not a primary focus of the trial; therefore, we could not exclude participants based on cognitive impairment at baseline. However, our rigorous screening procedures effectively excluded those with clear impairment, and randomization facilitated comparable demographic and health characteristics across study arms at baseline. Therefore, there is no reason to suspect that the two groups would have differed in cognitive performance at baseline had it been measured. Our findings are generalizable to only a high-risk subset of the population, i.e., older adults with diabetes and overweight/obesity. However, this group, a growing segment of the population, has an increased risk of cognitive impairment, making this work valuable for individuals with diabetes and overweight/obesity. A strength of the work is the fact that Look AHEAD was a long-term randomized controlled clinical trial and was conducted using rigorous methods. Participants have been closely followed for nearly twenty years, providing deep phenotyping and well-characterized outcomes.

Our study adds to this body of literature by illustrating complex relationships between daytime sleepiness and cognitive performance among Look AHEAD participants who all had diabetes and overweight or obesity. Findings expand upon prior findings linking sleepiness to later amyloid deposition (12), and raise questions about the potential for daytime sleepiness (40) as an early indicator of cognitive decline, perhaps tied to atrophy in the locus coeruleus. Future longitudinal studies examining modifiable risk factors for dementia should include early measures of daytime sleepiness together with AD biomarkers to investigate these associations more thoroughly and determine their temporality. New targets for intervention that emerge early in the disease process are crucial to making advances in AD research.

## Supplemental Materials

Additional material Supplementary PDF file supplied by authors.

**Supplemental Table 1:** Baseline characteristics by inclusion/exclusion status

Baseline Characteristics	Overall	Inclusion Status		*p* **-value**
		Excluded	Included	
N	5145	3367	1778	
Age (Mean)	58.7 (6.8)	59.5 (7.0)	57.2 (6.3)	<0.001
45-54	1620 (31.5%)	940 (27.9%)	680 (38.2%)	<0.001
55-64	2651 (51.5%)	1720 (51.1%)	931 (52.4%)	
65-74	874 (17.0%)	707 (21.0%)	167 (9.4%)	
Gender (% female)	3063 (59.5%)	1978 (58.8%)	1085 (61.0%)	0.12
Race and Ethnicity (%)				0.07
African American	804 (15.6%)	541 (16.1%)	263 (14.8%)	
Non-Hispanic White	3252 (63.2%)	2116 (62.9%)	1136 (63.9%)	
Hispanic	680 (13.2%)	425 (12.6%)	255 (14.3%)	
Other	408 (7.9%)	284 (8.4%)	124 (7.0%)	
Education (%)				<0.001
<13 years	1020 (20.3%)	693 (21.3%)	327 (18.4%)	
13-16 years	1916 (38.1%)	1270 (39.1%)	646 (36.3%)	
>16 years	2094 (41.6%)	1289 (39.6%)	805 (45.3%)	
*APOE* ε4 carrier (%)	969 (23.5%)	584 (24.9%)	385 (21.6%)	0.02
Body Mass Index (Mean)	35.9 (5.9)	35.9 (5.8)	36.1 (6.0)	0.30
25-29	765 (14.9%)	503 (14.9%)	262 (14.7%)	0.53
30-39	3231 (62.8%)	2128 (63.2%)	1103 (62.1%)	
≥40	1149 (22.3%)	736 (21.9%)	413 (23.2%)	
History of CVD (%)	712 (13.8%)	544 (16.2%)	168 (9.5%)	<0.001
Hypertension (%)	4281 (83.2%)	2847 (84.6%)	1434 (80.7%)	<0.001
Insulin use (%)	795 (16.0%)	540 (17.0%)	255 (14.3%)	0.02
Diabetes duration, years (Mean)	6.8 (6.5)	6.9 (6.6)	6.5 (6.4)	0.04
HbA1c % (Mean)	7.28 (1.17)	7.31 (1.18)	7.23 (1.15)	0.02
Smoking status (%)				0.49
Current	227 (4.4%)	147 (4.4%)	80 (4.5%)	
Former	2331 (45.4%)	1544 (46.0%)	787 (44.3%)	
Never	2576 (50.2%)	1665 (49.6%)	911 (51.2%)	
Depressive symptoms (%)				0.04
BDI score <11	4474 (87.0%)	2904 (86.2%)	1570 (88.3%)	
BDI score ≥11	671 (13.0%)	463 (13.8%)	208 (11.7%)	
Daytime sleepiness (%)				0.80
Never	2312 (45.1%)	1526 (45.6%)	786 (44.2%)	
Sometimes	1654 (32.3%)	1073 (32.0%)	581 (32.7%)	
Often	667 (13.0%)	428 (12.8%)	239 (13.4%)	
Almost Always	496 (9.7%)	324 (9.7%)	172 (9.7%)	

Abbreviations: *APOE* ϵ4=Apolipoprotein E gene, ϵ4 carrier status; BDI=Beck Depression Inventory; CVD=cardiovascular disease; DSE=diabetes support and education; ILI=intensive lifestyle intervention; SD=standard deviation.

**Supplemental Table 2.** Cognitive Scores at 2018-2020 visit by 2013-2014 levels of Daytime Sleepiness [LS Mean (SE)] not adjusted for prior cognitive scores.

Outcome	Daytime Sleepiness at 2013-2014			
	Never	Sometimes	Often / Always	*p* -value*
Composite z-score	-0.58 (0.05)	-0.58 (0.04)	-0.68 (0.05)	0.06
3MS z-score	-0.72 (0.07)	-0.69 (0.06)	-0.68 (0.07)	0.82
Stroop z-score	-0.59 (0.08)	-0.56 (0.07)	-0.71 (0.08)	0.13
DSC z-score	**-0.76 (0.06)**	**-0.77 (0.05)**	**-0.93 (0.06)**	**0.01**
Trails-A z-score	-0.47 (0.07)	-0.55 (0.07)	-0.61 (0.07)	0.10
Trails-B z-score	**-0.58 (0.06)**	**-0.62 (0.06)**	**-0.73 (0.07)**	**0.05**
RAVLT Delayed z-score	-0.16 (0.06)	-0.15 (0.06)	-0.27 (0.06)	0.17

Abbreviations: 3MS=Modified Mini-mental State Exam; DSC=Digit Symbol Coding; RAVLT Delayed=Rey Auditory Verbal Learning Test Delayed; Trails A=Trail Making Test Part A; Trails B=Trail Making Test Part B. *Models are adjusted for arm, depressive symptoms at baseline, 2013-2014 and 2018-2020 visits, and baseline values of daytime sleepiness, age, gender, race and ethnicity, education, BMI, and hypertension.

**Supplemental Table 3.** Cognitive Scores at 2018-2020 visit by Daytime Sleepiness at 2013-2014 Stratified by Intervention Arm [LS Mean (SE)] not adjusted for prior cognitive scores.

Intervention Arm	Outcome	Daytime Sleepiness at 2013-2014			
		Never	Sometimes	Often / Always	*p* -value*
DSE	Composite z-score	-0.58 (0.06)	-0.52 (0.06)	-0.66 (0.06)	0.08
	3MS z-score	-0.69 (0.09)	-0.67 (0.09)	-0.72 (0.10)	0.86
	Stroop z-score	-0.56 (0.11)	-0.48 (0.11)	-0.72 (0.11)	0.10
	DSC z-score	-0.75 (0.08)	-0.68 (0.08)	-0.87 (0.08)	0.07
	Trails-A z-score	-0.38 (0.09)	-0.38 (0.09)	-0.46 (0.09)	0.66
	Trails-B z-score	-0.56 (0.09)	-0.51 (0.08)	-0.61 (0.09)	0.54
	RAVLT Delayed z-score	-0.24 (0.09)	-0.16 (0.09)	-0.28 (0.10)	0.37

ILI	Composite z-score	-0.59 (0.07)	-0.64 (0.06)	-0.71 (0.07)	0.22
	3MS z-score	-0.75 (0.10)	-0.72 (0.09)	-0.65 (0.10)	0.58
	Stroop z-score	-0.63 (0.11)	-0.64 (0.11)	-0.71 (0.11)	0.75
	DSC z-score	**-0.77 (0.08)**	**-0.87 (0.08)**	**-0.99 (0.08)**	**0.02**
	Trails-A z-score	-0.56 (0.10)	-0.71 (0.10)	-0.75 (0.10)	0.08
	Trails-B z-score	**-0.60 (0.09)**	**-0.73 (0.09)**	**-0.86 (0.09)**	**0.02**
	RAVLT Delayed z-score	-0.10 (0.09)	-0.15 (0.09)	-0.27 (0.09)	0.19

Abbreviations: 3MS=Modified Mini-mental State Exam; DSC=Digit Symbol Coding; DSE=Diabetes Support and Education Arm; ILI=Intensive Lifestyle Intervention Arm; RAVLT Delayed=Rey Auditory Verbal Learning Test Delayed; Trails A=Trail Making Test Part A; Trails B=Trail Making Test Part B. *Stratified models are adjusted for depressive symptoms at baseline, 2013-2014 and 2018-2020 visits, and baseline values of daytime sleepiness, age, gender, race and ethnicity, education, BMI, and hypertension.

**Supplemental Table 4.** Interactions between baseline characteristics and cognitive scores* [LS Mean (SE)]

Characteristic	Outcome	Daytime Sleepiness at 2013-2014			
		Never	Sometimes	Often/Always	*p* **-value**
**Race and Ethnicity**					
African American	**Cognitive Composite**	-0.29 (0.05)	-0.35 (0.05)	-0.33 (0.07)	0.01
Non-Hispanic White		-0.22 (0.03)	-0.16 (0.03)	-0.32 (0.03)	
Hispanic		-0.24 (0.05)	-0.35 (0.05)	-0.22 (0.06)	
Other		-0.40 (0.08)	-0.27 (0.06)	-0.35 (0.10)	

African American	**Stroop**	-0.63 (0.10)	-0.51 (0.11)	-0.65 (0.15)	0.03
Non-Hispanic White		-0.33 (0.07)	-0.23 (0.07)	-0.45 (0.07)	
Hispanic		-0.49 (0.11)	-0.87 (0.11)	-0.54 (0.15)	
Other		-0.47 (0.16)	-0.24 (0.13)	-0.51 (0.21)	

African American	**DSC**	-0.52 (0.06)	-0.51 (0.07)	-0.56 (0.09)	0.02
Non-Hispanic White		-0.43 (0.04)	-0.35 (0.04)	-0.52 (0.04)	
Hispanic		-0.47 (0.07)	-0.52 (0.06)	-0.33 (0.08)	
Other		-0.44 (0.10)	-0.52 (0.07)	-0.38 (0.12)	

African American	**Trails A**	-0.43 (0.09)	-0.32 (0.10)	-0.58 (0.14)	0.09
Non-Hispanic White		-0.14 (0.06)	-0.23 (0.06)	-0.29 (0.06)	
Hispanic		-0.49 (0.10)	-0.41 (0.10)	-0.20 (0.12)	
Other		-0.25 (0.15)	-0.42 (0.11)	-0.34 (0.19)	

African American	**Trails B**	-0.25 (0.09)	-0.48 (0.09)	-0.46 (0.13)	0.08
Non-Hispanic White		-0.15 (0.06)	-0.13 (0.06)	-0.30 (0.06)	
Hispanic		-0.31 (0.09)	-0.39 (0.09)	-0.43 (0.12)	
Other		-0.53 (0.14)	-0.60 (0.11)	-0.19 (0.18)	

African American	**RAVLT Delayed**	-0.12 (0.08)	-0.30 (0.09)	-0.03 (0.12)	0.07
Non-Hispanic White		0.03 (0.05)	0.07 (0.05)	-0.07 (0.06)	
Hispanic		0.15 (0.09)	0.02 (0.08)	0.04 (0.11)	
Other		-0.16 (0.13)	-0.02 (0.10)	-0.39 (0.17)	
*APOE* ** ε4 Status**					
1 or 2 ε4 alleles	**Trails A**	-0.46 (0.08)	-0.29 (0.08)	-0.44 (0.11)	0.05
No ε4 alleles		-0.27 (0.06)	-0.38 (0.06)	-0.38 (0.06)	

1 or 2 ε4 alleles	**RAVLT Delayed**	-0.06 (0.07)	-0.23 (0.07)	-0.15 (0.09)	0.08
No ε4 alleles		-0.02 (0.05)	0.03 (0.05)	-0.10 (0.05)	
**Baseline History of CVD**					
No	**Stroop**	-0.45 (0.07)	-0.46 (0.06)	-0.59 (0.07)	<0.01
Yes		-0.69 (0.13)	-0.12 (0.15)	-0.43 (0.15)	

No	**Trails A**	-0.32 (0.06)	-0.34 (0.06)	-0.36 (0.06)	0.08
Yes		-0.26 (0.11)	-0.48 (0.13)	-0.68 (0.14)	

No	**Trails B**	-0.31 (0.06)	-0.38 (0.05)	-0.42 (0.06)	0.09
Yes		-0.41 (0.11)	-0.22 (0.12)	-0.63 (0.13)	
**Baseline Depressive Symptoms**					
BDI < 11	**DSC**	-0.49 (0.04)	-0.47 (0.04)	-0.58 (0.04)	<0.01
BDI ≥ 11		-0.59 (0.08)	-0.44 (0.07)	-0.35 (0.07)	

BDI < 11	**Trails A**	-0.33 (0.06)	-0.32 (0.06)	-0.43 (0.06)	<0.01
BDI ≥ 11		-0.20 (0.12)	-0.59 (0.10)	-0.22 (0.11)	

BDI < 11	**Trails B**	-0.36 (0.06)	-0.39 (0.05)	-0.53 (0.06)	0.02
BDI ≥ 11		-0.37 (0.11)	-0.44 (0.10)	-0.20 (0.10)	

Abbreviations: BDI=Beck Depression Inventory; BMI=body mass index; CVD=cardiovascular disease; DSC=Digit Symbol Coding; LS=least squares; RAVLT=Rey Auditory Learning Test; SE=standard error; Trails A=Trail Making Test Part A; Trails B=Trail Making Test Part B.

## Coordinating Center

Wake Forest University Mark A. Espeland, PhD1; Kathleen Hayden, PhD^1^; Judy L. Bahnson, BA, CCRP^3^; Lynne E. Wagenknecht, DrPH^3^; David Reboussin, PhD^3^; Nicholas Pajewski, PhD^3^; Jingzhong Ding, PhD^3^; Gagan Deep, PhD^3^; Stephen R. Rapp, PhD^3^; Bonnie C. Sachs, PhD^3^; Jerry M. Barnes, MA; Tara D. Beckner; Delilah R. Cook; Joni Evans, MS; Katie Garcia, MS; Sarah A. Gaussoin, MS; Carol Kittel, MS; Lea Harvin, BS; Marjorie Howard, MS; Rebecca H. Neiberg, MS; Jennifer Walker, MS; Michael P. Walkup, MS. ^1^Principal Investigator; ^2^Program Coordinator; ^3^Co-Investigator; All other Look AHEAD staffs are listed alphabetically by site.
