# Supplementary material for: Daytime Sleepiness Is Associated with Lower Cognitive Scores: The Look AHEAD Study
Source: JAR Life. 2023 Jun 26;12:46–55. doi: 10.14283/jarlife.2023.9 (PMC10345450; doi:10.14283/jarlife.2023.9)
Supplement: Additional material — Supplementary PDF file supplied by authors. [file jarlife-12-009-S1.docx]

**Supplemental Materials**

**Supplemental Table 1: Baseline characteristics by inclusion/exclusion status**

| **Baseline Characteristics** | **Overall** | **Inclusion Status** | | ***p*-value** |
| --- | --- | --- | --- | --- |
|  |  | **Excluded** | **Included** |  |
| N | 5145 | 3367 | 1778 |  |
| Age (Mean) | 58.7 (6.8) | 59.5 (7.0) | 57.2 (6.3) | <0.001 |
| 45-54 | 1620 (31.5%) | 940 (27.9%) | 680 (38.2%) | <0.001 |
| 55-64 | 2651 (51.5%) | 1720 (51.1%) | 931 (52.4%) |  |
| 65-74 | 874 (17.0%) | 707 (21.0%) | 167 (9.4%) |  |
| Gender (% female) | 3063 (59.5%) | 1978 (58.8%) | 1085 (61.0%) | 0.12 |
| Race and Ethnicity (%) |  |  |  | 0.07 |
| African American | 804 (15.6%) | 541 (16.1%) | 263 (14.8%) |  |
| Non-Hispanic White | 3252 (63.2%) | 2116 (62.9%) | 1136 (63.9%) |  |
| Hispanic | 680 (13.2%) | 425 (12.6%) | 255 (14.3%) |  |
| Other | 408 (7.9%) | 284 (8.4%) | 124 (7.0%) |  |
| Education (%) |  |  |  | <0.001 |
| <13 years | 1020 (20.3%) | 693 (21.3%) | 327 (18.4%) |  |
| 13-16 years | 1916 (38.1%) | 1270 (39.1%) | 646 (36.3%) |  |
| >16 years | 2094 (41.6%) | 1289 (39.6%) | 805 (45.3%) |  |
| *APOE* ε4 carrier (%) | 969 (23.5%) | 584 (24.9%) | 385 (21.6%) | 0.02 |
| Body Mass Index (Mean) | 35.9 (5.9) | 35.9 (5.8) | 36.1 (6.0) | 0.30 |
| 25-29 | 765 (14.9%) | 503 (14.9%) | 262 (14.7%) | 0.53 |
| 30-39 | 3231 (62.8%) | 2128 (63.2%) | 1103 (62.1%) |  |
| ≥40 | 1149 (22.3%) | 736 (21.9%) | 413 (23.2%) |  |
| History of CVD (%) | 712 (13.8%) | 544 (16.2%) | 168 (9.5%) | <0.001 |
| Hypertension (%) | 4281 (83.2%) | 2847 (84.6%) | 1434 (80.7%) | <0.001 |
| Insulin use (%) | 795 (16.0%) | 540 (17.0%) | 255 (14.3%) | 0.02 |
| Diabetes duration, years (Mean) | 6.8 (6.5) | 6.9 (6.6) | 6.5 (6.4) | 0.04 |
| HbA1c % (Mean) | 7.28 (1.17) | 7.31 (1.18) | 7.23 (1.15) | 0.02 |
| Smoking status (%) |  |  |  | 0.49 |
| Current | 227 (4.4%) | 147 (4.4%) | 80 (4.5%) |  |
| Former | 2331 (45.4%) | 1544 (46.0%) | 787 (44.3%) |  |
| Never | 2576 (50.2%) | 1665 (49.6%) | 911 (51.2%) |  |
| Depressive symptoms (%) |  |  |  | 0.04 |
| BDI score <11 | 4474 (87.0%) | 2904 (86.2%) | 1570 (88.3%) |  |
| BDI score ≥11 | 671 (13.0%) | 463 (13.8%) | 208 (11.7%) |  |
| Daytime sleepiness (%) |  |  |  | 0.80 |
| Never | 2312 (45.1%) | 1526 (45.6%) | 786 (44.2%) |  |
| Sometimes | 1654 (32.3%) | 1073 (32.0%) | 581 (32.7%) |  |
| Often | 667 (13.0%) | 428 (12.8%) | 239 (13.4%) |  |
| Almost Always | 496 (9.7%) | 324 (9.7%) | 172 (9.7%) |  |

Abbreviations: *APOE* ε4=Apolipoprotein E gene, ε4 carrier status; BDI=Beck Depression Inventory; CVD=cardiovascular disease; DSE=diabetes support and education; ILI=intensive lifestyle intervention; SD=standard deviation.

**Supplemental Table 2. Cognitive Scores at 2018-2020 visit by 2013-2014 levels of Daytime Sleepiness [LS Mean (SE)] not adjusted for prior cognitive scores.**

| **Outcome** | **Daytime Sleepiness at 2013-2014** | | | |
| --- | --- | --- | --- | --- |
|  | **Never** | **Sometimes** | **Often / Always** | ***p*-value*** |
| Composite z-score | -0.58 (0.05) | -0.58 (0.04) | -0.68 (0.05) | 0.06 |
| 3MS z-score | -0.72 (0.07) | -0.69 (0.06) | -0.68 (0.07) | 0.82 |
| Stroop z-score | -0.59 (0.08) | -0.56 (0.07) | -0.71 (0.08) | 0.13 |
| DSC z-score | **-0.76 (0.06)** | **-0.77 (0.05)** | **-0.93 (0.06)** | **0.01** |
| Trails-A z-score | -0.47 (0.07) | -0.55 (0.07) | -0.61 (0.07) | 0.10 |
| Trails-B z-score | **-0.58 (0.06)** | **-0.62 (0.06)** | **-0.73 (0.07)** | **0.05** |
| RAVLT Delayed z-score | -0.16 (0.06) | -0.15 (0.06) | -0.27 (0.06) | 0.17 |

Abbreviations: 3MS=Modified Mini-mental State Exam; DSC=Digit Symbol Coding; RAVLT Delayed=Rey Auditory Verbal Learning Test Delayed; Trails A=Trail Making Test Part A; Trails B=Trail Making Test Part B.

*Models are adjusted for arm, depressive symptoms at baseline, 2013-2014 and 2018-2020 visits, and baseline values of daytime sleepiness, age, gender, race and ethnicity, education, BMI, and hypertension.

**Supplemental Table 3. Cognitive Scores at 2018-2020 visit by Daytime Sleepiness at 2013-2014 Stratified by Intervention Arm [LS Mean (SE)] not adjusted for prior cognitive scores.**

| **Intervention Arm** | **Outcome** | **Daytime Sleepiness at 2013-2014** | | | |
| --- | --- | --- | --- | --- | --- |
|  |  | **Never** | **Sometimes** | **Often / Always** | ***p*-value*** |
| DSE | Composite z-score | -0.58 (0.06) | -0.52 (0.06) | -0.66 (0.06) | 0.08 |
|  | 3MS z-score | -0.69 (0.09) | -0.67 (0.09) | -0.72 (0.10) | 0.86 |
|  | Stroop z-score | -0.56 (0.11) | -0.48 (0.11) | -0.72 (0.11) | 0.10 |
|  | DSC z-score | -0.75 (0.08) | -0.68 (0.08) | -0.87 (0.08) | 0.07 |
|  | Trails-A z-score | -0.38 (0.09) | -0.38 (0.09) | -0.46 (0.09) | 0.66 |
|  | Trails-B z-score | -0.56 (0.09) | -0.51 (0.08) | -0.61 (0.09) | 0.54 |
|  | RAVLT Delayed z-score | -0.24 (0.09) | -0.16 (0.09) | -0.28 (0.10) | 0.37 |
|  |  |  |  |  |  |
| ILI | Composite z-score | -0.59 (0.07) | -0.64 (0.06) | -0.71 (0.07) | 0.22 |
|  | 3MS z-score | -0.75 (0.10) | -0.72 (0.09) | -0.65 (0.10) | 0.58 |
|  | Stroop z-score | -0.63 (0.11) | -0.64 (0.11) | -0.71 (0.11) | 0.75 |
|  | DSC z-score | **-0.77 (0.08)** | **-0.87 (0.08)** | **-0.99 (0.08)** | **0.02** |
|  | Trails-A z-score | -0.56 (0.10) | -0.71 (0.10) | -0.75 (0.10) | 0.08 |
|  | Trails-B z-score | **-0.60 (0.09)** | **-0.73 (0.09)** | **-0.86 (0.09)** | **0.02** |
|  | RAVLT Delayed z-score | -0.10 (0.09) | -0.15 (0.09) | -0.27 (0.09) | 0.19 |

Abbreviations: 3MS=Modified Mini-mental State Exam; DSC=Digit Symbol Coding; DSE=Diabetes Support and Education Arm; ILI=Intensive Lifestyle Intervention Arm; RAVLT Delayed=Rey Auditory Verbal Learning Test Delayed; Trails A=Trail Making Test Part A; Trails B=Trail Making Test Part B.

*Stratified models are adjusted for depressive symptoms at baseline, 2013-2014 and 2018-2020 visits, and baseline values of daytime sleepiness, age, gender, race and ethnicity, education, BMI, and hypertension.

**Supplemental Table 4. Interactions between baseline characteristics and cognitive scores* [LS Mean (SE)]**

| **Characteristic** | **Outcome** | **Daytime Sleepiness at 2013-2014** | | | |
| --- | --- | --- | --- | --- | --- |
|  |  | **Never** | **Sometimes** | **Often/Always** | ***p*-value** |
| **Race and Ethnicity** | | | | | |
| African American | **Cognitive Composite** | -0.29 (0.05) | -0.35 (0.05) | -0.33 (0.07) | 0.01 |
| Non-Hispanic White |  | -0.22 (0.03) | -0.16 (0.03) | -0.32 (0.03) |  |
| Hispanic |  | -0.24 (0.05) | -0.35 (0.05) | -0.22 (0.06) |  |
| Other |  | -0.40 (0.08) | -0.27 (0.06) | -0.35 (0.10) |  |
|  |  |  |  |  |  |
| African American | **Stroop** | -0.63 (0.10) | -0.51 (0.11) | -0.65 (0.15) | 0.03 |
| Non-Hispanic White |  | -0.33 (0.07) | -0.23 (0.07) | -0.45 (0.07) |  |
| Hispanic |  | -0.49 (0.11) | -0.87 (0.11) | -0.54 (0.15) |  |
| Other |  | -0.47 (0.16) | -0.24 (0.13) | -0.51 (0.21) |  |
|  |  |  |  |  |  |
| African American | **DSC** | -0.52 (0.06) | -0.51 (0.07) | -0.56 (0.09) | 0.02 |
| Non-Hispanic White |  | -0.43 (0.04) | -0.35 (0.04) | -0.52 (0.04) |  |
| Hispanic |  | -0.47 (0.07) | -0.52 (0.06) | -0.33 (0.08) |  |
| Other |  | -0.44 (0.10) | -0.52 (0.07) | -0.38 (0.12) |  |
|  |  |  |  |  |  |
| African American | **Trails A** | -0.43 (0.09) | -0.32 (0.10) | -0.58 (0.14) | 0.09 |
| Non-Hispanic White |  | -0.14 (0.06) | -0.23 (0.06) | -0.29 (0.06) |  |
| Hispanic |  | -0.49 (0.10) | -0.41 (0.10) | -0.20 (0.12) |  |
| Other |  | -0.25 (0.15) | -0.42 (0.11) | -0.34 (0.19) |  |
|  |  |  |  |  |  |
| African American | **Trails B** | -0.25 (0.09) | -0.48 (0.09) | -0.46 (0.13) | 0.08 |
| Non-Hispanic White |  | -0.15 (0.06) | -0.13 (0.06) | -0.30 (0.06) |  |
| Hispanic |  | -0.31 (0.09) | -0.39 (0.09) | -0.43 (0.12) |  |
| Other |  | -0.53 (0.14) | -0.60 (0.11) | -0.19 (0.18) |  |
|  |  |  |  |  |  |
| African American | **RAVLT Delayed** | -0.12 (0.08) | -0.30 (0.09) | -0.03 (0.12) | 0.07 |
| Non-Hispanic White |  | 0.03 (0.05) | 0.07 (0.05) | -0.07 (0.06) |  |
| Hispanic |  | 0.15 (0.09) | 0.02 (0.08) | 0.04 (0.11) |  |
| Other |  | -0.16 (0.13) | -0.02 (0.10) | -0.39 (0.17) |  |
| ***APOE* ε4 Status** | | | | | |
| 1 or 2 ε4 alleles | **Trails A** | -0.46 (0.08) | -0.29 (0.08) | -0.44 (0.11) | 0.05 |
| No ε4 alleles |  | -0.27 (0.06) | -0.38 (0.06) | -0.38 (0.06) |  |
|  | | | | | |
| 1 or 2 ε4 alleles | **RAVLT Delayed** | -0.06 (0.07) | -0.23 (0.07) | -0.15 (0.09) | 0.08 |
| No ε4 alleles |  | -0.02 (0.05) | 0.03 (0.05) | -0.10 (0.05) |  |
| **Baseline History of CVD** | | | | | |
| No | **Stroop** | -0.45 (0.07) | -0.46 (0.06) | -0.59 (0.07) | <0.01 |
| Yes |  | -0.69 (0.13) | -0.12 (0.15) | -0.43 (0.15) |  |
|  | | | | | |
| No | **Trails A** | -0.32 (0.06) | -0.34 (0.06) | -0.36 (0.06) | 0.08 |
| Yes |  | -0.26 (0.11) | -0.48 (0.13) | -0.68 (0.14) |  |
|  | | | | | |
| No | **Trails B** | -0.31 (0.06) | -0.38 (0.05) | -0.42 (0.06) | 0.09 |
| Yes |  | -0.41 (0.11) | -0.22 (0.12) | -0.63 (0.13) |  |
| **Baseline Depressive Symptoms** | | | | | |
| BDI < 11 | **DSC** | -0.49 (0.04) | -0.47 (0.04) | -0.58 (0.04) | <0.01 |
| BDI ≥ 11 |  | -0.59 (0.08) | -0.44 (0.07) | -0.35 (0.07) |  |
|  | | | | | |
| BDI < 11 | **Trails A** | -0.33 (0.06) | -0.32 (0.06) | -0.43 (0.06) | <0.01 |
| BDI ≥ 11 |  | -0.20 (0.12) | -0.59 (0.10) | -0.22 (0.11) |  |
|  | | | | | |
| BDI < 11 | **Trails B** | -0.36 (0.06) | -0.39 (0.05) | -0.53 (0.06) | 0.02 |
| BDI ≥ 11 |  | -0.37 (0.11) | -0.44 (0.10) | -0.20 (0.10) |  |

Abbreviations: BDI=Beck Depression Inventory; BMI=body mass index; CVD=cardiovascular disease; DSC=Digit Symbol Coding; LS=least squares; RAVLT=Rey Auditory Learning Test; SE=standard error; Trails A=Trail Making Test Part A; Trails B=Trail Making Test Part B.
